# Nur77-deficiency in bone marrow-derived macrophages modulates inflammatory responses, extracellular matrix homeostasis, phagocytosis and tolerance

**DOI:** 10.1186/s12864-016-2469-9

**Published:** 2016-03-01

**Authors:** Anouk A. J. Hamers, Carmen Argmann, Perry D. Moerland, Duco S. Koenis, Goran Marinković, Milka Sokolović, Alex F. de Vos, Carlie J. M. de Vries, Claudia M. van Tiel

**Affiliations:** Department of Medical Biochemistry, Academic Medical Center, University of Amsterdam, Amsterdam, The Netherlands; Present address: Department of Inflammation Biology, La Jolla Institute for Allergy and Immunology, San Diego, USA; Present address: Institute for Genomics and Multiscale Biology Mount Sinai Hospital, New York, USA; Bioinformatics Laboratory, Department of Clinical Epidemiology, Biostatistics and Bioinformatics, Academic Medical Center, University of Amsterdam, Amsterdam, The Netherlands; Present address: European Food Information Council, Brussels, Belgium; Center for Experimental and Molecular Medicine, Academic Medical Center, University of Amsterdam, Amsterdam, The Netherlands

**Keywords:** Nur77, TR3, NR4A1, Macrophage, Collagen, Inflammation, SDF-1α, Rac1, CX3CR1

## Abstract

**Background:**

The nuclear orphan receptor Nur77 (NR4A1, TR3, or NGFI-B) has been shown to modulate the inflammatory response of macrophages. To further elucidate the role of Nur77 in macrophage physiology, we compared the transcriptome of bone marrow-derived macrophages (BMM) from wild-type (WT) and Nur77-knockout (KO) mice.

**Results:**

In line with previous observations, SDF-1α (CXCL12) was among the most upregulated genes in Nur77-deficient BMM and we demonstrated that Nur77 binds directly to the SDF-1α promoter, resulting in inhibition of SDF-1α expression. The cytokine receptor CX3CR1 was strongly downregulated in Nur77-KO BMM, implying involvement of Nur77 in macrophage tolerance. Ingenuity pathway analyses (IPA) to identify canonical pathways regulation and gene set enrichment analyses (GSEA) revealed a potential role for Nur77 in extracellular matrix homeostasis. Nur77-deficiency increased the collagen content of macrophage extracellular matrix through enhanced expression of several collagen subtypes and diminished matrix metalloproteinase (MMP)-9 activity. IPA upstream regulator analyses discerned the small GTPase Rac1 as a novel regulator of Nur77-mediated gene expression. We identified an inhibitory feedback loop with increased Rac1 activity in Nur77-KO BMM, which may explain the augmented phagocytic activity of these cells. Finally, we predict multiple chronic inflammatory diseases to be influenced by macrophage Nur77 expression. GSEA and IPA associated Nur77 to osteoarthritis, chronic obstructive pulmonary disease, rheumatoid arthritis, psoriasis, and allergic airway inflammatory diseases.

**Conclusions:**

Altogether these data identify Nur77 as a modulator of macrophage function and an interesting target to treat chronic inflammatory disease.

**Electronic supplementary material:**

The online version of this article (doi:10.1186/s12864-016-2469-9) contains supplementary material, which is available to authorized users.

## Background

Nuclear receptor Nur77, also known as NR4A1, TR3 or NGFI-B, is a member of the NR4A receptor subfamily that also comprises Nurr1 (NR4A2, NOT) and NOR-1 (NR4A3, MINOR). Like other nuclear receptors, the NR4As consist of an N-terminal transactivation domain, a central zinc finger DNA binding domain (~94 % homology within the subfamily) and a C-terminal ligand binding domain. Structural analyses revealed that the NR4A receptors lack a classical hydrophobic ligand-binding pocket as a result of hydrophobic residues of amino-acid side chains, and so far no ligands have been identified [1, 2]. Nur77 is therefore referred to as an orphan nuclear receptor and its activity is regulated through gene expression, posttranslational modifications and coregulator interactions, as recently reviewed [3]. Nur77 is a typical early response gene and its induction can be achieved with a plethora of stimuli among which peptide hormones, mitogens, physical stimulation and inflammatory factors. Known transcription factors inducing its expression include cyclic adenosine monophosphate (cAMP) responsive element binding protein (CREB), activator protein 1 (AP-1), NFκB, and myocyte enhancer factor 2 (MEF2). Nur77 can bind as a monomer to the so-called NGFI-B response element (NBRE; AAAGGCTA) in the promoter region of direct target genes. Nur77 and Nurr1 can also form homodimers and heterodimers with retinoid X receptor and bind a DR-5 response element [4]. Furthermore, gene transcription is modulated by Nur77 itself through transrepression of other transcription factors. For example, Nur77 exhibits a direct, inhibitory interaction with the p65 subunit of NFκB [5, 6].

In macrophages, Nur77 is expressed in response to proinflammatory stimuli like prostaglandins, tumor necrosis factor-α (TNFα), lipopolysaccharide (LPS), interferon gamma (IFNγ) and granulocyte-macrophage colony stimulating factor (GM-CSF) [7, 8]. There may exist some discrepancy regarding Nur77’s anti-inflammatory role in macrophage function [7–13]. To elucidate the role of Nur77 in macrophages in more detail, we cultured bone marrow-derived macrophages (BMM) from wild-type (WT) and Nur77-deficient (Nur77-KO) mice, stimulated the cells with LPS and employed, to our knowledge for the first time, a gene expression study in these cells. Our data support functional involvement of Nur77 in (activated) macrophage physiology, by revealing the inhibition of stromal-derived factor (SDF)-1α expression, regulation of Rac1-mediated phagocytosis, extracellular matrix homeostasis and tolerance.

## Results

### Expression profiling reveals that Nur77 modulates inflammatory gene expression in macrophages

**Fig. 1:**
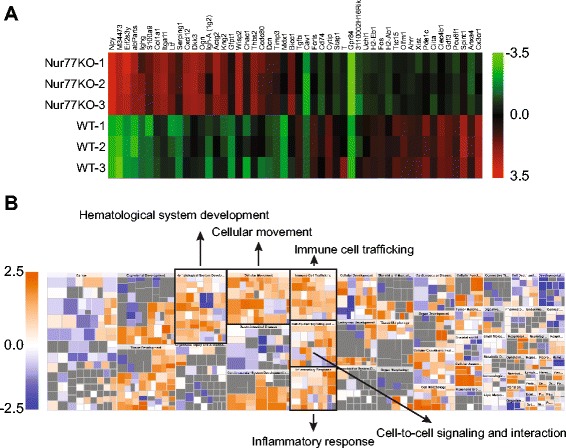
Nur77 modulates inflammatory gene expression in macrophages. **a** Heatmap of the top 25 up- and downregulated genes in Nur77-KO compared with WT BMM. The changes in mRNA abundance in Nur77-KO compared to WT BMM were determined by microarray analysis. The color-coded scale (*green* indicates downregulation and *red* indicates upregulation) for the log_2_-transformed expression values, normalized per gene, is indicated at the right of the figure. **b** Increased and decreased biological functions in Nu77-KO compared to WT BMM identified by IPA downstream effects analysis. The color-coded scale at the left of the figure reflects the direction of change for the function, based on the regulation z-score (*orange* indicates upregulation and *blue* indicates downregulation, *white* represents a z-score of zero and *grey* represents not available). The size of the box reflects the *p*-values (*large box*, small p-value)

To understand the function of Nur77 in macrophages, RNA was isolated from BMM isolates derived from Nur77-KO and WT mice and transcriptionally profiled. In these cells 324 genes were differentially expressed in Nur77-KO compared with WT BMM (*p*-value <0.05, absolute fold change ≥1.4), of which 64 % were upregulated and 36 % were downregulated in Nur77-KO compared with WT BMM. The top 25 up- and downregulated genes are shown in Fig. 1a and listed in Additional file 1: Table S1. These data were verified by qRT-PCR (Additional file 2: Figure S1) for S100 calcium binding protein A9 (S100A9), Neuropeptide Y (NPY) and serine (or cysteine) peptidase inhibitor G1 (Serping1) showing higher expression in Nur77-KO BMM and for FBJ osteosarcoma oncogene (cFos) showing decreased expression. We performed Ingenuity pathway downstream effects analysis to visualize the effect of Nur77 deficiency on biological processes and disease. Our microarray data imply enhanced inflammation in Nur77-KO BMM as inflammatory response, cell-to-cell signaling and interaction, hematological system development, cellular movement and immune cell trafficking are predicted to be activated in these cells compared to WT BMM (Fig. 1b).

### In BMM Nur77 reduces SDF-1α promoter activity

Previously, we have shown that SDF-1α, also known as CXCL12, mRNA expression is increased in Nur77-KO BMM, independent of stimulation and that overexpression of Nur77 in Nur77-KO BMM normalized SDF-1α expression [10]. In the current study, SDF-1α was also present in the top 25 of upregulated genes in Nur77-deficient BMM (Fig. 1 and Table S1).

**Fig. 2:**
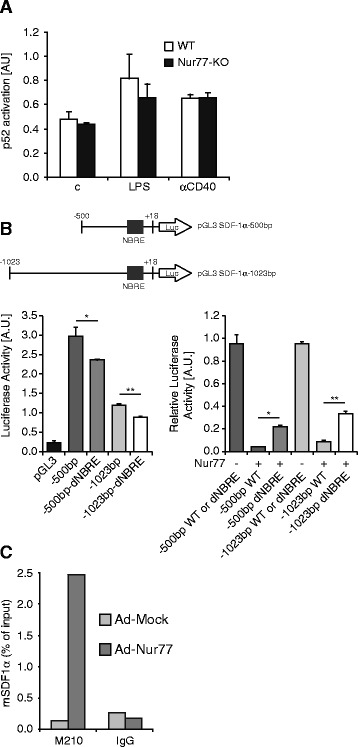
Nur77 represses SDF-1α expression through binding to an NBRE in the SDF-1α promoter. **a** The activation of the non-canonical NFκB pathway was measured in Nur77-KO and WT BMM by determining active p52 levels in the presence of LPS or CD40 activating antibody. **b** Analysis of the SDF-1α promoter region revealed the presence of an NBRE. Mutation analysis showed involvement of this NBRE in Nur77-dependent repression of SDF1α expression as measured by luciferase activity. Data were normalized for transfection efficiency by corresponding Renilla luciferase activity and in the right panel are depicted relative to the luciferase activity in the absence of Nur77. dNBRE, mutation of NBRE. **c** Nur77 binding to the SDF-1α promoter was determined by ChIP analyses using SDF-1α promoter-specific primers and Nur77-specific antibodies (M210) or control IgG in BMM after lentiviral overexpression of Nur77. Data are representative of at least three independent experiments performed in triplicate. Values represent mean ± S.D. *p < 0.05, **p < 0.01

Nur77-KO BMM have been shown to exhibit a proinflammatory phenotype after LPS activation involving enhanced expression of multiple cytokines [10, 12]. For many cytokines the enhanced expression in the absence of Nur77 may be explained by the transrepressive interaction between Nur77 and p65 NFκB [5, 6]. Induction of SDF-1α expression, however, involves activation of the non-canonical p52 NFκB pathway [14, 15]. In order to establish the mechanism by which Nur77 represses SDF-1α, we investigated p52 activity, which turned out to be similar in WT and Nur77-KO BMM upon LPS or CD40 activation. These data indicate that the difference in SDF-1α expression due to Nur77 deficiency cannot be explained by changes in the p52 NFκB signaling pathway (Fig. 2a). Subsequently, we analyzed the sequence of the SDF-1α promoter and found one potential NBRE consensus sequence at position -160 bp in the SDF-1α promoter region: ‘AAAGGTC*T*’ in which the last A residue of the consensus sequence is missing (Fig. 2b). Two promoter reporter constructs comprising bp −1023 to +18 and bp −500 to +18 were placed in luciferase reporter plasmids and were tested for activity. The shorter fragment drives higher basal luciferase expression than the longer construct, which may be caused by inhibitory elements in the bp −500 to −1023 region of the promoter. Overexpression of Nur77 resulted in strong inhibition of both reporters. Furthermore, mutation of the potential NBRE (AAAG*AA*CT; dNBRE) reduced this inhibitory activity of Nur77 4–5 fold (Fig. 2b). These data are in line with the data obtained from ChIP experiments revealing that Nur77 interacts with this SDF-1α promoter region (Fig. 2c).

### Nur77 enhances expression of CX3CR1 in BMM

**Fig. 3:**
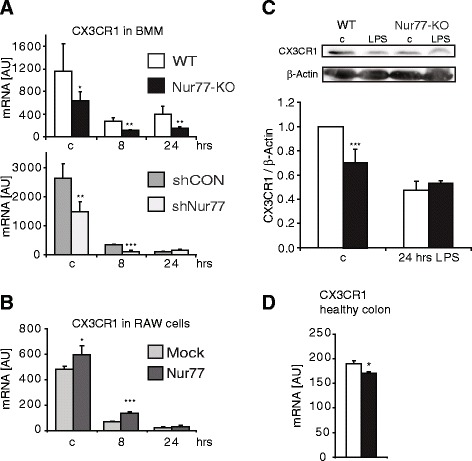
CX3CR1 is downregulated in Nur77-KO BMM and its expression is reduced in colons of Nur77-KO mice. mRNA expression of CX3CR1 was determined by qRT-PCR after treatment with 100 ng/ml LPS for the indicated time points in (**a**) WT and Nur77-KO BMM and in WT BMM transduced with control (shCON) or Nur77 (shNur77) shRNA and (**b**) in RAW cells transfected with control (Mock) or Nur77-encoding plasmids. **c** CX3CR1 protein expression in WT and Nur77-KO BMM after stimulation with 100 ng/ml LPS for 0 h (c) or 24 h. Protein expression was normalized to β-actin expression for quantification. **d** mRNA expression of CX3CR1 in colons of WT and Nur77-KO mice was determined by qRT-PCR. Data are expressed as mean ± S.D. *p < 0.05, **p < 0.01, ***p < 0.001

One of the most strongly downregulated genes in Nur77-KO BMM was CX3CR1, which is the receptor for the cytokine CX3CL1, also known as fractalkine. CX3CR1 is expressed on a subset of monocytes that in mice are typically Ly6C^lo^. These monocytes are known as patrolling monocytes with a crucial function in endothelial cell maintenance and are lacking in Nur77-KO mice [16]. qPCR confirmed decreased CX3CR1 mRNA expression in Nur77-KO BMM compared to WT cells (Fig. 3a). In line with this observation, Nur77 knockdown in WT BMM showed diminished CX3CR1 expression compared to shCON treated cells (Fig. 3a), whereas Nur77 overexpression in RAW cells resulted in increased CX3CR1 expression (Fig. 3b). Moreover, CX3CR1 protein expression was attenuated in Nur77-KO BMM compared to WT BMM (Fig. 3c). CX3CR1 deficiency in mice results in a decrease of lamina propria macrophages and increased severity of experimental colitis [17]. Since macrophage CX3CR1 plays such an important role in gut tolerance, we also determined its mRNA expression in mouse colon (Fig. 3d) and observed a modest but significant reduction of CX3CR1 expression in Nur77-KO colon lysates, which may indicate a decreased macrophage tolerance possibly making Nur77-KO mice more prone to develop colitis. We have recently investigated the function of Nur77 in inflammatory bowel disease and have shown that deficiency of Nur77 indeed aggravates both DSS- and TNBS-induced colitis [18].

### Modulation of extracellular matrix production in BMM by Nur77

**Fig. 4:**
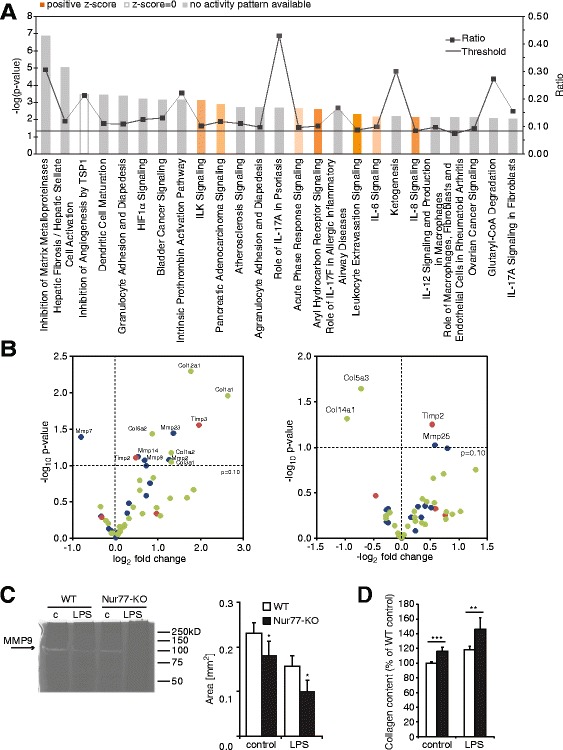
Nur77 deficiency results in decreased MMP activity and enhanced collagen content. **a** The top 25 canonical pathways associated with differentially expressed genes comparing Nur77-KO with WT BMMs were identified by IPA. The line graph represents the ratio of differentially expressed genes from the dataset to the genes present in each canonical pathway. The pathways are ranked from highest to lowest degree of association between genes from the data set with the pathways by the *p*-value, calculated by a right tailed Fisher Exact Test. The bar graph represents the -log(*p*-value) and the threshold represents *p* = 0.05. **b** The -log10 p-values of the MMPs (*blue*), TIMPs (*red*) and collagens (*green*) were plotted against their corresponding log2 fold changes (Nur77-KO vs WT) in unstimulated (*left panel*) and LPS stimulated (*right panel*) BMM. **c** MMP activity in BMM lysates derived from WT and Nur77-KO mice was determined by zymography and MMP9 activity was quantified (*right panel*). c, control; LPS, 24 h stimulation with 100 ng/ml LPS. **d** Collagen content measured in WT and Nur77-KO BMM stimulated for 24 h with 100 ng/ml LPS. *p < 0.05, **p < 0.01, ***p < 0.001

To obtain a better understanding of the functional differences between WT and Nur77-KO macrophages, we performed Ingenuity pathway analyses (IPA) to identify canonical pathways with a statistically significant enrichment of differentially expressed genes. The top 25 canonical pathways discriminating WT from Nur77-KO BMM are shown in Fig. 4a and details on the genes regulated in those pathways are presented in Additional file 1: Table S2. The top ranking canonical pathway is ‘Inhibition of matrix metalloproteinases’ (MMPs), which comprises 36 genes, 11 of which show changed expression in Nur77-KO BMM compared with WT cells. Interestingly, 13 of the top 25 canonical pathways involve regulation of MMPs, their inhibitors the tissue inhibitor of metalloproteinases (TIMPs) and/or collagens (Additional file 1: Table S2). Additionally, gene set enrichment analysis (GSEA) was performed to identify Biocarta, KEGG, PID or Reactome pathways that are regulated in Nur77-KO BMM compared to WT cells. Of the top 20 enriched gene sets, 12 are related to extracellular matrix interactions (Additional file 1: Table S3), involving collagens. Subsequently, the expression of all genes encoding proteins belonging to MMP, TIMP or collagen families was summarized in a Volcano plot (Fig. 4b), revealing that MMP7 expression was reduced in Nur77-KO BMM (0.6 fold, *p* = 0.04), whereas the expression of MMP2 (2.4-fold, *p* = 0.08), MMP9 (1.6-fold, *p* = 0.09), MMP14 (1.5-fold, *p* = 0.08), MMP23 (2.6-fold, *p* = 0.04), TIMP2 (1.4-fold, *p* = 0.08) and TIMP3 (4-fold, *p* = 0.03) was increased or showing a trend towards increased expression. MMPs and TIMPs are crucial regulators of extracellular matrix composition and macrophages have been shown to produce collagens [19–21]. The expression of several collagen subtypes (Col1a1, Col6a2 and Col12a1; 6-fold, 1.8-fold and 3.3-fold, respectively; *p* < 0.05) was significantly increased in Nur77-deficient BMM. Total MMP activity in protein lysates from Nur77-KO and WT BMM was determined by zymography, and as shown in Fig. 4c, both under control conditions as well as after LPS stimulation, especially MMP9 (92kD), was significantly lower in Nur77-KO BMM compared to WT cells. The balance between MMP- and TIMP activity and collagen synthesis eventually determines the net collagen content of the extracellular matrix. Because each of these aspects was changed in Nur77-KO BMM, the collagen content of BMM was subsequently measured and was significantly higher in Nur77-KO BMM both before and after LPS stimulation (Fig. 4d) compared to WT BMM. These data clearly reveal involvement of Nur77 in extracellular matrix composition, unexpectedly indicating that Nur77 reduces collagen content of BMM matrix.

### Disease pathways

To link gene sets that are upregulated in Nur77-KO BMM compared to WT cells with human disease pathways, we performed GSEA (Table 1). Several of these diseases are already known to be associated with Nur77, namely vascular disease (carotid artery disease and coronary disease), cancer (hematologic neoplasms, hepatocellular carcinoma), metabolism (glucose intolerance and diabetic nephropathies, angiopathies and retinopathy) and kidney ischemia reperfusion injury [10, 22–28]. Westbrook et al. [29] have shown that Nur77-deficient rats on a kidney injury-susceptible genetic background exhibited decreased renal function and attenuated kidney injury, which was rescued by bone marrow transplanted from control animals, suggesting a strong immune cell or even macrophage mediation. Furthermore, proteinuria was reported in Nur77-KO rats, which correlates with the current GSEA analysis predicting proteinuria. However, we did not find a difference in urine total protein or albumin levels between healthy WT and Nur77-KO mice, which of course may be different in kidney disease models (data not shown). In addition, our GSEA indicated that Nur77 may functionally be involved in systemic scleroderma; this is characterized by thickening of the skin caused by accumulation of collagen. Palumbo-Zerr et al. have recently shown that loss of Nur77 exacerbates fibrosis in multiple models of skin fibrosis [30], which is in line with the increased collagen production we observed in Nur77-KO BMM. GSEA indicated that Nur77 may also functionally be involved with macular degeneration, chronic obstructive pulmonary disease and osteoarthritis. The latter corresponds with the IPA revealing several rheumatoid arthritis signaling pathways to be effected (Fig. 4a and Additional file 1: Table S2).

**Table 1:** Top 20 human disease pathway gene sets identified with GSEA that are upregulated in Nur77-KO vs WT BMM

Human disease pathways	NES	*p*-value	Adjusted *p*-value	Molecules contributing to the pathway
Birth weight	2.060	0.002	0.007	NPY,H19,SERPINH1,GHR,IGF2
Diabetes mellitus type 1	1.993	<0.001	0.015	NPY,CXCL12,DCN,HP,CALD1,IGF2,TAF5L, RAGE,VWF,CBLB,ENPP1,VEGFA,AGER,VDR, SOD3
Proteinuria	1.856	<0.001	0.090	NPY,DCN,HP,ADM,RAGE,ENPP1,VEGFA,AGER
Diabetic nephropathies	1.854	0.002	0.068	DCN,HP,CCL5,MPO,CALD1,MMP9,ADM,RAGE,VWF,ENPP1,VEGFA,AGER,APOE
Pulmonary disease, chronic obstructive	1.852	0.007	0.057	COL1A1,MPO,SERPINE2,MMP9,GSTM1,TIMP2,VDR, SOD3
Carotid artery diseases	1.840	0.003	0.054	NPY,CXCL12,MMP3,MMP9
Coronary disease	1.826	0.003	0.058	NPY,PDGFRA,VLDLR,HP,COL3A1,MMP2, CDKN2B,CDKN2A,F7,MMP3,MMP9,F2R, HMGCR,VWF,ENPP1,VEGFA,APOE,VDR,LDLR,ESR1,SCARB1
Macular degeneration	1.818	<0.001	0.056	C2,HTRA1,MMP9,VEGFA,APOE,ERCC6
Fracture bone	1.804	0.004	0.058	COL1A1,COL1A2,TNFRSF11B,MMP9
Diabetic angiopathies	1.798	0.007	0.056	NPY,HP,F7,VWF,VEGFA,AGER
Diabetic retinopathy	1.791	0.009	0.055	NPY,RAGE,VEGFA,AGER
Scleroderma systemic	1.789	0.012	0.052	SPARC,FBN1,COL1A2,CCL5,FN1,IL1A
Glucose intolerance	1.789	0.011	0.048	NPY,GHR
Carcinoma ductal breast	1.788	0.005	0.045	MMP2,MMP9,ATM,VEGFA
Osteoarthritis	1.766	0.004	0.055	COL1A1,WISP1,MMP2,ENPP1,VDR,COL2A1
Carcinoma hepatocellular	1.736	0.011	0.072	HP,TFRC,F7,IGF2,HMGCR,APC,GSTM1
Osteoporosis, postmenopausal	1.724	0.012	0.078	COL1A1,TNFRSF11B,PLXNA2,VDR,ESR1, CALCA
Hematologic neoplasms	1.683	0.021	0.116	CXCL12,HPSE,GSTM1
Cognition disorders	1.678	0.019	0.117	VLDLR,MPO,BCHE,HMGCR,APOE,PRNP,DRD4, DTNBP1,CPOX
Angina, unstable	1.672	0.017	0.118	COL3A1,F7,MMP3,TAF1

*NES* Normalized enrichment score

### Upstream regulators

**Fig. 5:**
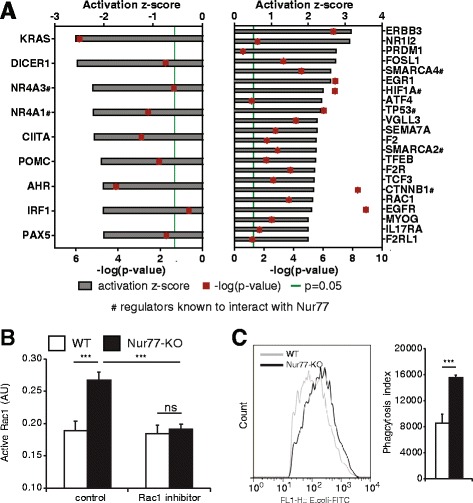
Rac1 is an upstream regulator activated in Nur77-KO BMM resulting in enhanced phagocytosis in these cells. **a** Upstream regulators in Nur77-KO compared to WT BMM identified by IPA upstream regulator analysis. The bias-corrected z-score and the *p*-value were calculated as described in Additional file 1: Table S4. **b** Active Rac1 levels were determined in the presence and absence of the Rac1 inhibitor 553502. **c** Phagocytosis of FITC-labeled uropathogenic *E. coli* bacteria by WT and Nur77-KO BMM was measured by flow cytometry and the phagocytosis index was calculated as the percentage of cells with internalized bacteria times the mean fluorescence intensity. Data are expressed as mean ± S.D. ns, not significant, ***p < 0.001

IPA Upstream Regulator Analysis was used to identify proteins that may be responsible for gene expression changes observed in our dataset. IPA predicts which upstream regulators are activated or inhibited to explain the upregulated and downregulated genes observed in the dataset. Knowledge of this regulatory cascade may indicate novel pathways that elucidate (part of) the observed gene expression changes in our dataset. IPA upstream regulator analysis identified 22 upstream regulators that are activated in Nur77-KO BMM and nine inhibited factors, among which Nur77 (NR4A1). Six of the upstream regulators, besides Nur77 itself, have been described to interact with Nur77; NOR1, SMARCA2, SMARCA4, TP53, HIF-1α, and β-catenin (CTNNB1), resulting in altered signaling [31] (Additional file 1: Table S4, Fig. 5a). In addition to transcription factors, also kinases and enzymes are among the predicted upstream regulators. Our analysis revealed Rac1 as an activated upstream regulator possibly regulating Nur77-mediated changes in gene expression. We therefore measured the levels of active Rac1 in Nur77-KO and WT BMM and observed that only Nur77-KO BMM contain active Rac1, which is completely inhibited by Rac1 inhibitor, whereas WT BMM show no basal Rac1 activity (Fig. 5b), indicating that Nur77 is involved in a negative feedback loop with Rac1. Since Rac1 is highly involved in cell motility and cytoskeleton movements in phagocytosis [32–34], we subsequently tested the Fcγ receptor-mediated phagocytosis capacity of Nur77-KO and WT BMM. As is shown in Fig. 5c, the phagocytosis index of Nur77-KO BMM regarding uropathogenic *E. coli* was almost 2-fold higher compared to WT cells. In addition to being involved in phagocytosis, Rac1 is known to inhibit MMP9 expression [35] correlating with our findings that MMP9 activity is lower in Nur77-KO BMM (Fig. 4c).

## Discussion

**Fig. 6:**
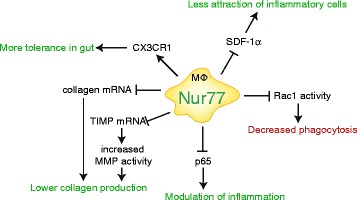
Role of Nur77 in macrophages. Nur77 modulates the inflammatory state of macrophages through multiple mechanisms, decreases collagen production by lowering TIMP1-3 and collagen mRNA expression, and lowers Rac1 activity resulting in decreased phagocytosis capacity. Green indicates a protective effect and red depicts a negative effect of Nur77 expression

In the current study we performed a transcriptome analysis to explore the function of Nur77 in macrophage function in addition to its known role in modulation of inflammatory responses [10, 12]. The obtained data uncovered involvement of Nur77 in extracellular matrix modulation, macrophage tolerance and inhibition of phagocytosis (schematically summarized in Fig. 6) and insight in its potential function in several diseases.

In line with our previous observations, CXCL12/SDF-1α expression was higher in Nur77-KO BMM than in WT cells. Even though Nur77 transrepresses NFκB p65, Nur77 did not affect NFκB p52 activity. Rather, direct binding of Nur77 to the SDF-1α promoter was shown to be crucial to inhibit expression of this gene. It was unexpected to detect diminished MMP9 activity, increased TIMP1-3 gene expression and enhanced expression of several collagen subtypes, among which Col1a1, present in the list of top-25 regulated genes, Col6a1 and Col12a1. Of note, Wang et al. [36] recently demonstrated that Nur77 augments MMP9 expression in colorectal cancer cells promoting invasion and metastasis of colorectal cancer. Corresponding with these observations, Nur77-KO BMM were shown to synthesize more collagen in their subcellular matrix compared to WT macrophages. This aspect of macrophages is relevant to wound healing, however, wound closure is normal in Nur77-KO mice [37]. Wound healing is a complex process involving multiple cell types, which may explain why overexpression of a dominant-negative variant of Nur77 in endothelial cells disturbs normal wound healing. In a similar way, Nur77 may need to be studied in more detail in macrophages during wound healing.

The most strongly downregulated gene in the absence of Nur77 was CX3CR1, which is especially expressed on colon lamina propria macrophages that sample antigen from the intestinal lumen [38]. CX3CR1^high^ macrophages produce IL-10, contributing to the maintenance and local expansion of protective regulatory T cells in the gut [39]. CX3CR1 expression was not only decreased in Nur77-KO BMM, but also in colon samples of these mice compared to WT colon. Previously, we have shown that IL-10 production by Nur77-KO BMM is lower compared to WT BMM [10]. Together, these data indicate a role for Nur77 in macrophage tolerance towards host bacteria in the gut and recently we have shown that dysregulation of Nur77 expression indeed leads to enhanced development of inflammatory bowel disease [18]. In the IPA upstream regulator analysis, we found the small GTPase Rac1 as an intriguing, unknown regulator of Nur77-mediated gene expression, which by itself is regulated by Nur77-deficiency. Rac1 is critically involved in cytoskeletal rearrangements [40, 41] and thus important in cell motility. In macrophages, it predominantly regulates Fcγ receptor-mediated phagocytosis [33, 34], cell fusion [42] and migration [43]. We found increased Rac1 activity upon Nur77 deficiency in BMM and a drastic increase in phagocytic capacity of Nur77-KO BMM in an *E.coli* opsonization model. These data strongly support our previous finding that Nur77-KO mice show a better initial clearance of bacteria during *E.coli*-induced peritonitis [11]. The latter study showed that the responsiveness of peritoneal macrophages to *E. coli* was only mildly affected by Nur77-deficiency, which is in line with our microarray data that reveal a limited effect of Nur77-deficiency on LPS-induced changes in gene expression (Additional file 1: Tables S5 and S6).

Our unbiased analyses of signaling pathways and diseases resulted in multiple hits relating to vascular disease and atherosclerosis. More specifically, gene sets identified with GSEA are; ‘Signaling by PDGF, Smooth muscle cell contractions, Sphingolipid metabolism and CXCR4 pathway’ [44–46] (Additional file 1: Table S3). In addition, GSEA revealed ‘carotid artery diseases’ and ‘coronary disease’ in the top 20 of human disease pathways, whereas ‘atherosclerosis signaling’ was found in IPA amongst the top 25 of canonical pathways associated with differentially expressed genes in Nur77-KO versus WT BMM (Fig. 4 and Additional file 1: Table S2). Altogether, the analyses on BMM gene expression support the protective function of Nur77 in atherosclerosis [10, 12, 24]. The other two members of the same subfamily of nuclear hormone receptors, Nurr1 and NOR-1, are also expressed in human atherosclerotic lesion macrophages and were shown to repress LPS-induced inflammatory response in THP-1 macrophages [7]. Similarly as Nur77, transplantation of bone marrow deficient for NOR-1 into atherosclerotic mice, aggravates the formation of atherosclerotic lesions [47].

Even though our gene expression analyses were performed in macrophages, a potential involvement of Nur77 in ‘diabetic nephropathy, angiopathy and retinopathy and glucose intolerance’ was predicted from GSEA (Table 1), which may emphasize the relative importance of macrophages in these pathologies. Macrophages are indeed known to contribute to the nephropathies [48–52], angiopathies [53, 54] and retinopathy [55, 56] seen in diabetes. Nur77 regulates hepatic glucose homeostasis [57] and Nur77-deficiency causes increased diet-induced insulin resistance [23]. Regarding the recent observation that lipid storage by adipose tissue macrophages regulates systemic glucose tolerance [58], it may be interesting to investigate glucose tolerance after a Nur77-deficient bone marrow transplantation into Ob/Ob mice.

Finally, we found multiple, often chronic inflammatory diseases to be potentially influenced by macrophage Nur77 expression. In most of these diseases macrophages are known to play a role. GSEA (Table 1) revealed osteoarthritis, chronic obstructive pulmonary disease and macular degeneration, whereas IPA (Additional file 1: Table S2) associated Nur77 to rheumatoid arthritis, psoriasis and allergic airway inflammatory diseases. Interestingly, recently Kurakula et al. [59] showed a protective role for Nur77 in ovalbumin-induced airway inflammation. Nur77 expression is elevated in synovial tissue, cartilage and prostaglandin E2 (PGE2) stimulated chondrocytes from patients with rheumatoid arthritis, psoriatic arthritis or osteoarthritis, making Nur77 an attractive target in rheumatic diseases [60–63]. In addition, T cell-specific Nur77 overexpression results in reduction of incidence and severity of collagen-induced arthritis by promoting activation-induced T cell apoptosis and inhibition of CollagenII-specific antibody production [64]. Interestingly, Nurr1 has already been shown to modulate psoriasis and rheumatoid arthritis [60, 61, 65]. The observed associations reveal potential, and so far speculative, connections between Nur77 and rheumatoid arthritis, macular degeneration, psoriasis and chronic obstructive pulmonary disease and we therefore propose that it may be relevant to study the role of Nur77 in these diseases.

## Conclusions

Our transcriptome analysis to explore the role of Nur77 in macrophage function uncovered involvement of Nur77 in extracellular matrix modulation, macrophage tolerance and inhibition of phagocytosis and insight in its potential function in several diseases. In summary, as schematically shown in Fig. 6, Nur77 modulates the inflammatory state of macrophages by decreasing inflammatory gene expression in macrophages via NFκB transrepression and positively regulating CX3CR1 expression. Nur77 directly represses SDF-1α secretion, which may result in less chemo-attraction of inflammatory cells. In addition, Nur77 inhibits the collagen content of the extracellular matrix of macrophages, diminishes Rac1 activity and reduces phagocytosis of these cells. All these effects can influence a plethora of inflammatory and metabolic diseases, making Nur77 an interesting factor to study and maybe even to target in treatment of chronic inflammatory disease.

## Methods

### Mice

Nur77-KO mice [66] on a C57BL/6 background were kindly provided by Prof B.R. Binder (Vienna, Austria). All animal experiments were approved by the Committee for Animal Welfare of the Amsterdam Medical Center and were carried out in compliance with guidelines issued by the Dutch government.

### Macrophage cell culture and transfection

Each batch of bone marrow cells was isolated from femurs and tibiae of three wild-type (WT) mice and three Nur77-KO mice as described [10]. In brief, cells were cultured in RPMI-1640 (GIBCO Invitrogen) with 100 U/ml penicillin/streptomycin (GIBCO Invitrogen), 10 % heat-inactivated fetal calf serum (FCS; GIBCO Invitrogen) and 15 % L929 conditioned medium (LCM) for 8 days to generate BMM. BMM were seeded at a density of 1.5 × 105 cells/cm2 24 h before stimulation. In WT BMM Nur77 was knocked-down by lentiviral transduction with a short hairpin (sh-)Nur77 cloned into a p156RRL-sinPPT-CMV-GFP-PRE/NheI vector by Bonta et al. [7] Lentiviral particles were produced as described previously [7] and BMM were transduced for 24 h with recombinant lentivirus. After transduction, cells were cultured for another 24 h and thereafter stimulated with 100 ng/ml LPS. shCON lentivirus was taken along as a control. Knockdown was confirmed by qPCR.

RAW264.7 cells were cultured in RPMI-1640 supplemented with 10 % FCS and penicillin/streptomycin and were transfected using Lipofectamine LTX (all Invitrogen) according to the manufacturer’s instructions.

### Microarray profiling

RNA was isolated from Nur77-KO and WT BMM using the Aurum total RNA isolation kit (BioRad) and samples were sent to ServiceXS (Leiden, The Netherlands) for further microarray processing. In brief, to assess the quality of the samples, the concentration of the RNA was determined using the Nanodrop ND1000 spectrophotometer. The Agilent Bioanalyzer was used to analyze the quality and integrity of the RNA samples. The Illumina TotalPrep-96 RNA Amplification Kit was used to generate biotin labeled (biotin-16-UTP) amplified cRNA and the obtained biotinylated cRNA samples were hybridized onto the Illumina MouseWG-6 v2 arrays. The samples were scanned using the Illumina iScan array scanner and the data retrieved using Illumina’s Genomestudio v. 2011.1 software.

### Microarray pre-processing and data analysis

Analyses were carried out with Bioconductor packages in the statistical software package R (version 2.14.0). Normexp-by-control background correction, quantile normalization, and log2 transformation [67] were performed on the Illumina sample and control probe profiles using the limma package (version 3.10.2). The arrayQualityMetrics package (version 3.10.0) was used to assess whether the microarray data were of good quality. Only probes detected (detection *p*-value < 0.05) on at least one array were included in the differential expression analysis. Gene-wise linear models were fitted using the limma package. Differential expression between the different conditions was assessed via a moderated *t*-test. The illuminaMousev2.db package (version 1.12.2) was used to update the probe annotation provided by Illumina. The data set with differentially expressed genes (nominal *p*-value < 0.05) was analyzed using Ingenuity Pathway Analysis (IPA, Ingenuity Systems) to test for enriched canonical pathways, to identify biological functions that are expected to be affected and to identify upstream transcriptional regulators. The background set for the gene enrichment analyses consisted of all genes that were detected on at least one array. Significance of enrichment was calculated using a right-tailed Fisher’s Exact Test.

Gene set enrichment analysis (GSEA; version 2.2.0 [68],) was performed using default options except for the following: the permutation type = Geneset; and the number of permutations = 1000 [69, 70]. The gene set collection c2.cp.v5.0.symbols.gmt which includes canonical pathways was queried in this analysis. The gene set to query human diseases was derived from [71] and GSEA was performed as described above except that the minimum number of genes in a gene set was set at 4.

### Plasmids

A construct containing amino-terminally myc-tagged Nur77 has been described before [3]. Two fragments of the murine SDF-1α promoter containing a putative NBRE (from −500 to +18 bp and from −1023 to +18 bp relative to the transcriptional start site) were cloned into the pGL3 basic vector (Promega) in front of the firefly luciferase gene. The mutant of the promoter constructs in which the putative NBRE was disrupted was generated by site-directed mutagenesis using the QuickChange site-directed mutagenesis method (Stratagene) according to the manufacturer's instruction using the following primer: dNBRE forw: 5’-ctgggaagatcaaagAActcagcacccagcgg-3’ and dNBRE rev: 5’-ccgctgggtgctgagTTctttgatcttcccag-3’. The nucleotides in capital letters represent the mutated nucleotides in the SDF-1α promoter. The construct was verified by sequencing and did not contain any other sequence variations.

### NFκB p52 activation/binding

BMM were stimulated with either 100 ng/ml LPS for 1 h or 25 μg/ml CD40 activating antibody FGK45 (Bioceros BV) for 3 h, protein lysates were harvested and the levels of active p52 were determined using the TransAM NFκB family kit (Active Motif) according to the manufacturer’s protocol. The absorbance was measured at OD450 nm in a microplate reader (EL808, Bio-Tek instruments).

### Luciferase reporter assays

RAW264.7 cells were transiently transfected with wild-type or mutant SDF-1α luciferase reporter plasmids together with pCMV-Myc-Nur77 or pCMV-mock. pRL-TK Renilla reporter plasmid (Promega) was co-transfected as an internal control. Luciferase activity measurements were performed using the dual-luciferase reporter assay system (Promega) and Glomax Multi detection system (Promega) according to the manufacturer’s instructions. Each experiment (in duplicate) was repeated at least three times.

### Chromatin immunoprecipitation assay (ChIP)

BMM were infected with Mock- or Nur77-adenovirus at a MOI of 100 for 4 h, and 28 h after infection the cells were harvested for ChIP analysis. ChIP assays were performed using the Magnify ChIP system (Invitrogen) according to the manufacturer’s instructions. The following primers were used to amplify the SDF-1α promoter in semi-quantitative real-time (qRT)PCR: 5’-GACTGTTTCGTCTCTCAGGTTC-3’ (sense) and 5’-GCTGAGACCTTTGATCTTCCCA-3’ (antisense).

### qRT-PCR

Total RNA was extracted using the Aurum™ Total RNA Mini Kit (Biorad) and cDNA was made from 500 ng RNA using iScript cDNA Synthesis kit (BioRad). qRT-PCR was performed using iQ SYBR Green Supermix (BioRad) and was measured using the MyIQ system and the following primers: Cx3cr1 forw: 5’-gagtatgacgattctgctgagg-3’ and Cx3cr1 rev: 5’-cagaccgaacgtgaagacgag-3’. Ribosomal protein 36B4 expression was determined to correct for cDNA content (36B4 forw: 5’-ggacccgagaagacctcctt-3’, 36B4 rev: 5’-gcacatcactcagaatttcaatgg-3’).

### Western blot analysis

BMM were treated for 24 h with 100 ng/ml LPS, after which the cells were washed twice with PBS and lysed in ice-cold Nonidet P-40 (NP-40) lysis buffer (50 mM Tris–HCl pH7.4, 100 mM NaCl, 10 mM NaF, 1 mM Na_3_PO_4_, 10 % glycerol, 1 % NP-40). After a 10 min incubation on ice the lysates were collected and boiled in sample buffer containing DTT. Samples were thereafter analyzed by SDS-PAGE. All proteins of interest were separated on 12 % polyacrylamide SDS gels. Proteins were transferred to 0.2 μm nitrocellulose membranes using the Trans-blot Turbo transfer system (Biorad). Nitrocellulose membranes were subsequently blocked in 5 % (w/v) non-fat milk in Tris-buffered saline (TBS) and incubated with CX3CR1 (Santa Cruz) and anti-α-tubulin (Cedarlane) primary antibodies overnight at 4 °C, followed by horse radish peroxidase-labelled secondary antibodies (Bio-Rad) for 1 h. Proteins were visualized with an enhanced chemiluminescence (ECL) detection system (Thermoscientific) and quantification of signal was performed using intensity measurements in ImageJ software.

### Collagen content assay

Collagen content of WT and Nur77-KO BMM was measured as described before [72]. Briefly, BMM were seeded at a density of 2 × 10^5^ cells/well in 24-wells plates. Cells were rinsed with PBS and fixed with 4 % para-formaldehyde and collagen content was stained by incubating with a 0.1 % (w/v) solution of Sirius Red F3B dye (BDH Laboratory Supplies) in 10 mM HCl for 40 min. Unbound dye was removed by washing extensively with 10 mM HCl, after which 100 mM NaOH was added to dissolve the dye and the absorbance was measured at OD550 nm in a microplate reader (EL808, Bio-Tek instruments).

### Zymography

Gelatinolytic metalloproteinase activity, especially MMP2 and MMP9, in WT and Nur77-KO BMM was measured by separating lysates of these cells on 10 % polyacrylamide gels containing gelatin (Ready-Gel zymogram gels, BioRad) under non-reducing conditions. After electrophoresis, the proteins were renatured by placing the gels in 2.5 % Triton X-100 for 30 min at room temperature and developed overnight in developing solution (50 mM Tris–HCl, pH 7.5, 200 mM NaCl, 5 mM CaCl2, 0.02 % Brij-35), followed by staining with PageBlue Protein Staining Solution (Thermo Scientific). After destaining with MilliQ, gelatinase activity was measured by densitometry.

### Rac1 activity assay

WT and Nur77-KO BMM were treated for 16 h with Rac1 inhibitor (553502; Calbiochem) followed by a 24-h incubation with 50 ng/ml interferon-γ (IFNγ, Peprotech). Cell lysates were prepared and the levels of Rac1-GTP were measured using a colorimetric-based G-LISA Rac1 (BK128; Cytoskeleton) activation assay according to the manufacturer’s protocol. OD490 nm was measured with a microplate reader (EL808, Bio-Tek instruments). Absorbance units in each sample were expressed after subtraction of the background units measured in protein-free lysis buffer.

### Phagocytosis

BMM were cultured overnight at 37 °C in RPMI containing 10 % FCS at a density of 0.5x10^6^ cells/well in 12-wells plates. BMM were washed twice with Hanks' balanced salt solution (GIBCO/Invitrogen) prior to the addition of fluorescently labeled bacteria. Uropathogenic *E. coli* (strain 1677) [73] were heat-killed by incubation at 65 °C for 1 h and labeled with 0.2 mg of FITC (Sigma-Aldrich) per ml in 0.1 M NaHCO_3_ (pH 9.0) for 1 h at 37 °C. The FITC-labeled *E. coli* (equivalent to 50 × 10^6^ CFU) were added to the BMM at a ratio of 100:1 and incubated for 1 h at 37 °C or 4 °C. Phagocytosis was stopped by immediately transferring the cells to 4 °C and washing them with ice-cold PBS containing 2 mM EDTA. The cells were harvested by gentle scraping and were subsequently treated with 0.4 % Trypan blue (GIBCO/Invitrogen) to quench extracellular fluorescence, washed twice with PBS/EDTA, and analyzed using a flow cytometer (Becton Dickinson FACScalibur). Results were expressed as phagocytosis index, defined as the percentage of cells with internalized *E. coli* times the mean fluorescence intensity.

### Statistical analysis

Data are reported as mean ± S.D. and were analyzed with the unpaired Student’s t test. Values of *p* < 0.05 were considered statistically significant (*p < 0.05, **p < 0.01, ***p < 0.001 in the figures).

### Availability of supporting data

The microarray data have been deposited in NCBI Gene Expression Omnibus in a MIAME compliant format and are accessible under GEO Series accession number GSE68167.

## Additional files

Additional file 1: Tables S1-S6.
**Table S1.** Top 25 up- and downregulated genes in Nur77-KO vs WT BMM. **Table S2.** Top 25 canonical pathways associated with differentially expressed genes in Nur77-KO vs WT BMM. **Table S3.** Top 20 gene sets identified with GSEA that are upregulated in Nur77-KO vs WT BMM. **Table S4.** Upstream Regulators in Nur77-KO vs WT BMM. **Table S5.** Top 25 up- and downregulated genes in LPS-stimulated Nur77-KO vs WT BMM. **Table S6.** Top 25 canonical pathways associated with differentially expressed genes in LPS-stimulated Nur77-KO vs WT BMM. (PDF 140 kb) Additional file 2: Figure S1. Verification of the microarray results by qRT-PCR. mRNA expression of top differentially expressed genes, as determined by microarray analysis, cFos, S100A9, NPY and Serping1 in WT and Nur77-KO BMM was determined by qRT-PCR before (left panel) and after (right panel) treatment with 100 ng/ml LPS. Data are expressed as mean ± S.D. **p* < 0.05, ***p* < 0.01, ****p* < 0.001, ns = not significant. (PDF 311 kb)

## References

[1] Flaig R, Greschik H, Peluso-Iltis C, Moras D (2005). Structural basis for the cell-specific activities of the NGFI-B and the Nurr1 ligand-binding domain. J Biol Chem.

[2] Wang Z, Benoit G, Liu J, Prasad S, Aarnisalo P, Liu X (2003). Structure and function of Nurr1 identifies a class of ligand-independent nuclear receptors. Nature.

[3] Kurakula K, van der Wal E, Geerts D, van Tiel CM, de Vries CJ (2011). FHL2 protein is a novel co-repressor of nuclear receptor Nur77. J Biol Chem.

[4] Pols TW, Bonta PI, de Vries CJ (2007). NR4A nuclear orphan receptors: protective in vascular disease?. Curr Opin Lipidol.

[5] Harant H, Lindley IJ (2004). Negative cross-talk between the human orphan nuclear receptor Nur77/NAK-1/TR3 and nuclear factor-kappaB. Nucleic Acids Res.

[6] Hong CY, Park JH, Ahn RS, Im SY, Choi HS, Soh J (2004). Molecular mechanism of suppression of testicular steroidogenesis by proinflammatory cytokine tumor necrosis factor alpha. Mol Cell Biol.

[7] Bonta PI, van Tiel CM, Vos M, Pols TW, van Thienen JV, Ferreira V (2006). Nuclear receptors Nur77, Nurr1, and NOR-1 expressed in atherosclerotic lesion macrophages reduce lipid loading and inflammatory responses. Arterioscler Thromb Vasc Biol.

[8] Shao Q, Shen LH, Hu LH, Pu J, Qi MY, Li WQ (2010). Nuclear receptor Nur77 suppresses inflammatory response dependent on COX-2 in macrophages induced by oxLDL. J Mol Cell Cardiol.

[9] Chao LC, Soto E, Hong C, Ito A, Pei L, Chawla A (2013). Bone marrow NR4A expression is not a dominant factor in the development of atherosclerosis or macrophage polarization in mice. J Lipid Res.

[10] Hamers AA, Vos M, Rassam F, Marinkovic G, Kurakula K, van Gorp PJ (2012). Bone marrow-specific deficiency of nuclear receptor Nur77 enhances atherosclerosis. Circ Res.

[11] Hamers AA, Uleman S, van Tiel CM, Kruijswijk D, van Stalborch AM, Huveneers S (2014). Limited role of nuclear receptor Nur77 in Escherichia coli-induced peritonitis. Infect Immun.

[12] Hanna RN, Shaked I, Hubbeling HG, Punt JA, Wu R, Herrley E (2012). NR4A1 (Nur77) deletion polarizes macrophages toward an inflammatory phenotype and increases atherosclerosis. Circ Res.

[13] Pei L, Castrillo A, Tontonoz P (2006). Regulation of macrophage inflammatory gene expression by the orphan nuclear receptor Nur77. Mol Endocrinol.

[14] Kew RR, Penzo M, Habiel DM, Marcu KB (2012). The IKKalpha-dependent NF-kappaB p52/RelB noncanonical pathway is essential to sustain a CXCL12 autocrine loop in cells migrating in response to HMGB1. J Immunol.

[15] McCorkell KA, May MJ (2015). Noncanonical NF-kappaB Activation and SDF-1 Expression in Human Endothelial Cells. Methods Mol Biol.

[16] Carlin LM, Stamatiades EG, Auffray C, Hanna RN, Glover L, Vizcay-Barrena G (2013). Nr4a1-dependent Ly6C(low) monocytes monitor endothelial cells and orchestrate their disposal. Cell.

[17] Medina-Contreras O, Geem D, Laur O, Williams IR, Lira SA, Nusrat A (2011). CX3CR1 regulates intestinal macrophage homeostasis, bacterial translocation, and colitogenic Th17 responses in mice. J Clin Invest.

[18] Hamers AA, van Dam L, Teixeira Duarte JM, Vos M, Marinković G, van Tiel CM (2015). Deficiency of Nuclear Receptor Nur77 Aggravates Mouse Experimental Colitis by Increased NFκB Activity in Macrophages. PLoS One.

[19] Schnoor M, Cullen P, Lorkowski J, Stolle K, Robenek H, Troyer D (2008). Production of type VI collagen by human macrophages: a new dimension in macrophage functional heterogeneity. J Immunol.

[20] Vaage J, Lindblad WJ (1990). Production of collagen type I by mouse peritoneal macrophages. J Leukoc Biol.

[21] Weitkamp B, Cullen P, Plenz G, Robenek H, Rauterberg J (1999). Human macrophages synthesize type VIII collagen in vitro and in the atherosclerotic plaque. FASEB J.

[22] Balasubramanian S, Jansen M, Valerius MT, Humphreys BD, Strom TB (2012). Orphan nuclear receptor Nur77 promotes acute kidney injury and renal epithelial apoptosis. J Am Soc Nephrol.

[23] Chao LC, Wroblewski K, Zhang Z, Pei L, Vergnes L, Ilkayeva OR (2009). Insulin resistance and altered systemic glucose metabolism in mice lacking Nur77. Diabetes.

[24] Hu YW, Zhang P, Yang JY, Huang JL, Ma X, Li SF (2014). Nur77 decreases atherosclerosis progression in apoE(−/−) mice fed a high-fat/high-cholesterol diet. PLoS One.

[25] Mullican SE, Zhang S, Konopleva M, Ruvolo V, Andreeff M, Milbrandt J (2007). Abrogation of nuclear receptors Nr4a3 and Nr4a1 leads to development of acute myeloid leukemia. Nat Med.

[26] Ramirez-Herrick AM, Mullican SE, Sheehan AM, Conneely OM (2011). Reduced NR4A gene dosage leads to mixed myelodysplastic/myeloproliferative neoplasms in mice. Blood.

[27] Yang H, Zhan Q, Wan YJ (2011). Enrichment of Nur77 mediated by retinoic acid receptor beta leads to apoptosis of human hepatocellular carcinoma cells induced by fenretinide and histone deacetylase inhibitors. Hepatology.

[28] Zhao BX, Chen HZ, Du XD, Luo J, He JP, Wang RH (2011). Orphan receptor TR3 enhances p53 transactivation and represses DNA double-strand break repair in hepatoma cells under ionizing radiation. Mol Endocrinol.

[29] Westbrook L, Johnson AC, Regner KR, Williams JM, Mattson DL, Kyle PB (2014). Genetic Susceptibility and Loss of Nr4a1 Enhances Macrophage-Mediated Renal Injury in CKD. J Am Soc Nephrol..

[30] Palumbo-Zerr K, Zerr P, Distler A, Fliehr J, Mancuso R, Huang J (2015). Orphan nuclear receptor NR4A1 regulates transforming growth factor-beta signaling and fibrosis. Nat Med.

[31] Kurakula K, Koenis DS, van Tiel CM, de Vries CJ (1843). NR4A nuclear receptors are orphans but not lonesome. Biochim Biophys Acta.

[32] Caron E, Hall A (1998). Identification of two distinct mechanisms of phagocytosis controlled by different Rho GTPases. Science.

[33] Cox D, Chang P, Zhang Q, Reddy PG, Bokoch GM, Greenberg S (1997). Requirements for both Rac1 and Cdc42 in membrane ruffling and phagocytosis in leukocytes. J Exp Med.

[34] Hoppe AD, Swanson JA (2004). Cdc42, Rac1, and Rac2 display distinct patterns of activation during phagocytosis. Mol Biol Cell.

[35] Murthy S, Ryan A, He C, Mallampalli RK, Carter AB (2010). Rac1-mediated mitochondrial H2O2 generation regulates MMP-9 gene expression in macrophages via inhibition of SP-1 and AP-1. J Biol Chem.

[36] Wang JR, Gan WJ, Li XM, Zhao YY, Li Y, Lu XX (2014). Orphan nuclear receptor Nur77 promotes colorectal cancer invasion and metastasis by regulating MMP-9 and E-cadherin. Carcinogenesis..

[37] Niu G, Ye T, Qin L, Bourbon PM, Chang C, Zhao S (2014). Orphan nuclear receptor TR3/Nur77 improves wound healing by upregulating the expression of integrin beta4. FASEB J..

[38] Mowat AM (2003). Anatomical basis of tolerance and immunity to intestinal antigens. Nat Rev Immunol.

[39] Bain CC, Scott CL, Uronen-Hansson H, Gudjonsson S, Jansson O, Grip O (2013). Resident and pro-inflammatory macrophages in the colon represent alternative context-dependent fates of the same Ly6Chi monocyte precursors. Mucosal Immunol.

[40] Kaibuchi K, Kuroda S, Amano M (1999). Regulation of the cytoskeleton and cell adhesion by the Rho family GTPases in mammalian cells. Annu Rev Biochem.

[41] Evers EE, Zondag GC, Malliri A, Price LS, ten Klooster JP, van der Kammen RA (2000). Rho family proteins in cell adhesion and cell migration. Eur J Cancer.

[42] Helming L, Gordon S (2009). Molecular mediators of macrophage fusion. Trends Cell Biol.

[43] Rose DM, Alon R, Ginsberg MH (2007). Integrin modulation and signaling in leukocyte adhesion and migration. Immunol Rev.

[44] Chatterjee S (1998). Sphingolipids in atherosclerosis and vascular biology. Arterioscler Thromb Vasc Biol.

[45] Shi W, Wang X, Wong J, Hedrick CC, Wong H, Castellani LW (2004). Effect of macrophage-derived apolipoprotein E on hyperlipidemia and atherosclerosis of LDLR-deficient mice. Biochem Biophys Res Commun.

[46] Weigert A, Weis N, Brune B (2009). Regulation of macrophage function by sphingosine-1-phosphate. Immunobiology.

[47] Qing H, Liu Y, Zhao Y, Aono J, Jones KL, Heywood EB (2014). Deficiency of the NR4A orphan nuclear receptor NOR1 in hematopoietic stem cells accelerates atherosclerosis. Stem Cells.

[48] Castiglione RC, Maron-Gutierrez T, Barbosa CM, Ornellas FM, Barreira AL, Dibarros CB (2013). Bone marrow-derived mononuclear cells promote improvement in glomerular function in rats with early diabetic nephropathy. Cell Physiol Biochem.

[49] Kuwabara T, Mori K, Mukoyama M, Kasahara M, Yokoi H, Nakao K (2014). Macrophage-mediated glucolipotoxicity via myeloid-related protein 8/toll-like receptor 4 signaling in diabetic nephropathy. Clin Exp Nephrol.

[50] Tesch G, Sourris KC, Summers SA, McCarthy D, Ward MS, Borg DJ (2014). Deletion of bone-marrow-derived receptor for AGEs (RAGE) improves renal function in an experimental mouse model of diabetes. Diabetologia.

[51] You H, Gao T, Cooper TK, Brian RW, Awad AS (2013). Macrophages directly mediate diabetic renal injury. Am J Physiol Renal Physiol.

[52] Zhang XL, Guo YF, Song ZX, Zhou M (2014). Vitamin D Prevents Podocyte Injury Via Regulation Of Macrophage M1/M2 Phenotype In Diabetic Nephropathy Rats. Endocrinology..

[53] Fadini GP, de Kreutzenberg SV, Boscaro E, Albiero M, Cappellari R, Krankel N (2013). An unbalanced monocyte polarisation in peripheral blood and bone marrow of patients with type 2 diabetes has an impact on microangiopathy. Diabetologia.

[54] Kanter JE, Bornfeldt KE (2013). Inflammation and diabetes-accelerated atherosclerosis: myeloid cell mediators. Trends Endocrinol Metab.

[55] Marchetti V, Yanes O, Aguilar E, Wang M, Friedlander D, Moreno S (2011). Differential macrophage polarization promotes tissue remodeling and repair in a model of ischemic retinopathy. Sci Rep.

[56] Urbancic M, Kloboves PV, Petrovic D, Globocnik PM (2013). A flow cytometric analysis of vitreous inflammatory cells in patients with proliferative diabetic retinopathy. Biomed Res Int.

[57] Kim YD, Kim SG, Hwang SL, Choi HS, Bae JH, Song DK (2014). B-cell translocation gene 2 regulates hepatic glucose homeostasis via induction of orphan nuclear receptor Nur77 in diabetic mouse model. Diabetes.

[58] Aouadi M, Vangala P, Yawe JC, Tencerova M, Nicoloro SM, Cohen JL (2014). Lipid storage by adipose tissue macrophages regulates systemic glucose tolerance. Am J Physiol Endocrinol Metab.

[59] Kurakula K, Vos M, Logiantara A, Roelofs JJ, Nieuwenhuis MA, Koppelman GH (2015). Nuclear Receptor Nur77 Attenuates Airway Inflammation in Mice by Suppressing NF-kappaB Activity in Lung Epithelial Cells. J Immunol.

[60] McEvoy AN, Murphy EA, Ponnio T, Conneely OM, Bresnihan B, Fitzgerald O (2002). Activation of nuclear orphan receptor NURR1 transcription by NF-kappa B and cyclic adenosine 5'-monophosphate response element-binding protein in rheumatoid arthritis synovial tissue. J Immunol.

[61] McMorrow JP, Murphy EP (2011). Inflammation: a role for NR4A orphan nuclear receptors?. Biochem Soc Trans.

[62] Mix KS, Attur MG, Al-Mussawir H, Abramson SB, Brinckerhoff CE, Murphy EP (2007). Transcriptional repression of matrix metalloproteinase gene expression by the orphan nuclear receptor NURR1 in cartilage. J Biol Chem.

[63] Murphy EP, McEvoy A, Conneely OM, Bresnihan B, Fitzgerald O (2001). Involvement of the nuclear orphan receptor NURR1 in the regulation of corticotropin-releasing hormone expression and actions in human inflammatory arthritis. Arthritis Rheum.

[64] De Silva S, Han S, Zhang X, Huston DP, Winoto A, Zheng B (2005). Reduction of the incidence and severity of collagen-induced arthritis by constitutive Nur77 expression in the T cell lineage. Arthritis Rheum.

[65] Ralph JA, Ahmed AU, Santos LL, Clark AR, McMorrow J, Murphy EP (2010). Identification of NURR1 as a mediator of MIF signaling during chronic arthritis: effects on glucocorticoid-induced MKP1. Am J Pathol.

[66] Lee SL, Wesselschmidt RL, Linette GP, Kanagawa O, Russell JH, Milbrandt J (1995). Unimpaired thymic and peripheral T cell death in mice lacking the nuclear receptor NGFI-B (Nur77). Science.

[67] Shi W, Oshlack A, Smyth GK (2010). Optimizing the noise versus bias trade-off for Illumina whole genome expression BeadChips. Nucleic Acids Res.

[68] Gene Set Enrichment Analysis at the Broad Institute of MIT and Harvard. http://www.broad.mit.edu/gsea.

[69] Mootha VK, Lindgren CM, Eriksson KF, Subramanian A, Sihag S, Lehar J (2003). PGC-1alpha-responsive genes involved in oxidative phosphorylation are coordinately downregulated in human diabetes. Nat Genet.

[70] Subramanian A, Tamayo P, Mootha VK, Mukherjee S, Ebert BL, Gillette MA (2005). Gene set enrichment analysis: a knowledge-based approach for interpreting genome-wide expression profiles. Proc Natl Acad Sci U S A.

[71] Zhang Y, De S, Garner JR, Smith K, Wang SA, Becker KG (2010). Systematic analysis, comparison, and integration of disease based human genetic association data and mouse genetic phenotypic information. BMC Med Genomics.

[72] Groenendijk BC, Benus GF, Klous A, Pacheco YM, Volger OL, Fledderus JO (2011). Activin A induces a non-fibrotic phenotype in smooth muscle cells in contrast to TGF-beta. Exp Cell Res.

[73] Elkahwaji JE, Ott CJ, Janda LM, Hopkins WJ (2005). Mouse model for acute bacterial prostatitis in genetically distinct inbred strains. Urology.

