# Supplementary material for: Nur77-deficiency in bone marrow-derived macrophages modulates inflammatory responses, extracellular matrix homeostasis, phagocytosis and tolerance
Source: BMC Genomics. 2016 Mar 1;17:162. doi: 10.1186/s12864-016-2469-9 (PMC4774191; doi:10.1186/s12864-016-2469-9)
Supplement: Additional file 1: Tables S1-S6. — Table S1. Top 25 up- and downregulated genes in Nur77-KO vs WT BMM. Table S2. Top 25 canonical pathways associated with differentially expressed genes in Nur77-KO vs WT BMM. Table S3. Top 20 gene sets identified with GSEA that are upregulated in Nur77-KO vs WT BMM. Table S4. Upstream Regulators in Nur77-KO vs WT BMM. Table S5. Top 25 up- and downregulated genes in LPS-stimulated Nur77-KO vs WT BMM. Table S6. Top 25 canonical pathways associated with differentially expressed genes in LPS-stimulated Nur77-KO vs WT BMM. (PDF 140 kb) [file 12864_2016_2469_MOESM1_ESM.pdf]

Table S1. Top 25 up- and downregulated genes in Nur77-KO vs WT BMM

Gene Symbol	Log2 Fold Change (KO/WT)	p-value	Entrez Gene ID
Upregulated genes			
Npy	5.6062	4.07E-04	109648
M34473	5.4207	9.67E-05	243469
Eif2s3y	4.6783	8.92E-04	26908
abParts	4.2134	5.56E-04	16142
Ighg	2.8760	1.14E-05	16019
S100a9	2.8396	1.62E-03	20202
Col1a1	2.6363	1.11E-02	12842
Itga11	2.6357	2.18E-03	319480
Ltf	2.5876	5.07E-03	17002
Serping1	2.5568	1.96E-02	12258
Cxcl12	2.5079	2.00E-02	20315
Dkk3	2.3926	2.97E-02	50781
Ogn	2.3816	6.60E-03	18295
Igh-A (1g2)	2.3501	2.30E-02	16061
Actg2	2.3012	5.30E-02	11468
Kng2	2.2874	1.42E-03	385643
Gfpt1	2.2597	4.73E-06	14583
Wisp2	2.2514	2.50E-03	22403
Chac1	2.2337	7.28E-02	69065
Thbs2	2.0130	9.57E-02	21826
Ccdc80	2.0016	8.76E-03	67896
Dcn	1.9975	9.20E-02	13179
Timp3	1.9637	2.82E-02	21859
Mxd1	1.9573	1.71E-03	17119
Bicc1	1.9430	1.18E-03	83675
Downregulated genes			
Tgfb1	-1.0752	0.12214	21810
Cav	-1.0835	0.01927	12389
Fcrls	-1.0933	0.00038	80891
Cd74	-1.1044	0.00368	16149
Cytip	-1.1077	0.00286	227929
Stap1	-1.1086	0.04858	56792
T	-1.1582	0.00591	20997
Gpr84	-1.1593	0.00394	80910
3110002H16Rik	-1.1687	0.04292	76482
Uchl1	-1.1945	0.06995	22223
H2-Eb1	-1.3139	0.00026	14969
Fos	-1.3209	0.01022	14281
H2-Ab1	-1.3297	0.00025	14961
Ttc15	-1.3564	0.01063	217449
Olfm1	-1.3627	0.07107	56177
Ahrr	-1.3669	0.00426	11624
Xist	-1.4229	0.02179	213742
Pde1c	-1.4947	0.02739	18575
Ciita	-1.5196	0.01751	12265
Clec4b1	-1.5515	0.00063	69810
Gdf3	-1.6907	0.00001	14562
Pou6f1	-1.7861	0.00009	19009
Spint1	-1.8194	0.00188	20732
Anxa4	-1.9455	0.00001	11746
Cx3cr1	-2.4795	<0.00001	13051

Table S2. Top 25 canonical pathways associated with differentially expressed genes in Nur77-KO vs WT BMM

IPA canonical pathway	p-value	Adjusted p-value	Ratio	z-score	Genes
**Inhibition of Matrix Metalloproteinases**	1.45E-07	5.89E-05	0.31	n/a	HSPG2,TIMP3,MMP7,MMP23B,SDC1,SDC2, MMP14,THBS2,MMP2,MMP9, TIMP2
**Hepatic Fibrosis / Hepatic Stellate Cell Activation**	1.00E-05	0.002	0.12	n/a	VCAM1,FN1,COL6A2,COL12A1,FGFR2, VEGFB,MMP2,IGFBP5,PDGFC,COL1A2, VEGFA,COL1A1,IGF2,IGF1,CD40,TGFB3, CD14,EDNRA,MMP9,TNFSF14,TIMP2, COL3A1
**Inhibition of Angiogenesis by TSP1**	3.55E-04	0.033	0.21	0.000	VEGFA,HSPG2,SDC1,SDC2,MAPK10,AKT3, MMP9
Dendritic Cell Maturation	3.80E-04	0.033	0.11	n/a	CREB3L4,HLA-DQB1,CREB5,DDR1,COL1A2, TRAF6,PIK3R3,COL1A1,CD40,HLA-DMA, IL1RN,FSCN1,MAPK10, AKT3,HLA-DRB5, COL3A1
**Granulocyte Adhesion and Diapedesis**	4.37E-04	0.033	0.11	n/a	ITGB1,VCAM1,MMP7,SDC1,Ppbp,MMP14, CXCL12,MMP2,MMP23B,ITGAM,ICAM2, Ccl8,SDC2,IL1RN,MMP9,HSPB1
**HIF1α Signaling**	0.001	0.033	0.13	n/a	VEGFA,PIK3R3,MMP7,MMP23B,MMP14, MAPK10,AKT3,VEGFB,MMP2,TCEB1, PDGFC,MMP9
**Bladder Cancer Signaling**	0.001	0.033	0.13	n/a	VEGFA,CDKN2A,MMP7,MMP23B,MMP14, FGF14,VEGFB,MMP2,PDGFC, MMP9,FGF13
Intrinsic Prothrombin Activation Pathway	0.001	0.033	0.22	n/a	KNG1,COL1A2,COL1A1,KLK3,F8, COL3A1
**ILK Signaling**	0.001	0.033	0.10	1.500	ITGB1,SNAI2,FN1,VEGFB,CREB3L4,CREB5, RICTOR,PDGFC,VEGFA,PIK3R3,FOS, PPAP2B,IRS1,MAPK10,AKT3, ACTG2,MMP9
**Pancreatic Adenocarcinoma Signaling**	0.001	0.050	0.12	1.265	VEGFA,CDKN2A,PIK3R3,HMOX1,MAPK10, TGFB3,AKT3,VEGFB,E2F3,NOTCH1,PDGFC, MMP9
**Atherosclerosis Signaling**	0.002	0.068	0.11	n/a	COL1A2,COL1A1,VCAM1,CD40,IL1RN, CXCL12,ALOX12,S100A8,PDGFC,TNFSF14, MMP9,COL3A1
**Agranulocyte Adhesion and Diapedesis**	0.002	0.068	0.10	n/a	ITGB1,AOC3,VCAM1,MMP7,FN1,Ppbp, MMP14,CXCL12,MMP2,MMP23B,ICAM2, Ccl8,IL1RN,ACTG2,MMP9
Role of IL-17A in Psoriasis	0.002	0.068	0.43	n/a	S100A9,IL17RC,S100A8
Acute Phase Response Signaling	0.002	0.068	0.10	0.905	SOCS3,SERPING1,FN1,CP,TRAF6,PIK3R3, HMOX1,FOS,HP,F8,IL1RN,AKT3OSMR, CRABP2,C2
Aryl Hydrocarbon Receptor Signaling	0.003	0.069	0.10	1.890	AHRR,GSTA3,CDKN2A,FOS,GSTM1,CCND2, NFIC,ALDH1L2,GSTM5,NQO1,TGFB3,NFIB, HSPB1
Role of IL-17F in Allergic Inflammatory Airway Diseases	0.003	0.089	0.17	n/a	TRAF6,RPS6KA6,IGF1,IL17RC,CREB3L4, CREB5
**Leukocyte Extravasation Signaling**	0.005	0.116	0.09	1.604	ITGB1,TIMP3,VCAM1,ARHGAP6,MMP7, MMP14,CXCL12,RAPGEF3,MMP2,PIK3R3, MMP23B,ITGAM,MAPK10,ACTG2,MMP9, TIMP2
IL-6 Signaling	0.007	0.139	0.10	1.000	VEGFA,TRAF6,PIK3R3,COL1A1,SOCS3,FOS, IL1RN,MAPK10,AKT3,CD14,HSPB1

Ketogenesis	0.007	0.139	0.30	n/a	ACAT2,HMGCS1,HADHA
**IL-8 Signaling**	0.007	0.139	0.08	1.807	VCAM1,VEGFB,MMP2,PDGFC,VEGFA, TRAF6,PIK3R3,FOS,HMOX1,ITGAM,CCND2, MPO,MAPK10,AKT3,MMP9
IL-12 Signaling and Production in Macrophages	0.008	0.139	0.10	n/a	TRAF6,PIK3R3,FOS,Ifna4,CD40,MAPK10, TGFB3,AKT3,ALOX12,S100A8,IFNA4
Role of Macrophages, Fibroblasts and Endothelial Cells in Rheumatoid Arthritis	0.008	0.139	0.07	n/a	SOCS3,VCAM1,FN1,SFRP2,DAAM1,GNAQ, CXCL12,IL17RC,VEGFB, CREB3L4,CREB5,PDGFC,TRAF6,VEGFA, PIK3R3,FOS,IL1RN,DKK3,TLR7,AKT3, NFATC2
**Ovarian Cancer Signaling**	0.008	0.139	0.09	n/a	VEGFA,CDKN2A,PIK3R3,GJA1,MMP7,PTGS1 AKT3,VEGFB,EDNRA,MMP2, PDGFC,MMP9
Glutaryl-CoA Degradation	0.009	0.158	0.27	n/a	ACAT2,HADHA,HSD17B8
IL-17A Signaling in Fibroblasts	0.010	0.162	0.16	n/a	TRAF6,NFKBID,FOS,LCN2,IL17RC

The pathways in bold involve matrix metalloproteinases (MMPs); red indicates upregulation, green indicates downregulation; n/a: not available

Table S3. Top 20 gene sets identified with GSEA that are upregulated in Nur77-KO vs WT BMM

Biocarta, KEGG, PID and Reactome pathways	NES	p-value	Adjusted p-value	Genes contributing to the pathway
**Collagen formation (Reactome)**	2.441	<0.001	<0.001	COL1A1,PCOLCE,COL12A1,COL3A1,COL1A2,COL5A1, COL6A1,COL6A2,COL16A1,COL4A2,BMP1,SERPINH1, PCOLCE2,COL4A1,ADAMTS2,COL14A1,PLOD2,COL6A3, COL8A1,COL5A2,COL17A1
**Extracellular matrix organization (Reactome)**	2.411	<0.001	<0.001	COL1A1,PCOLCE,COL12A1,COL3A1,COL1A2,COL5A1, COL6A1,COL6A2,MMP2,COL16A1,COL4A2,BMP1, SERPINH1,MMP3,PCOLCE2,COL4A1,ADAMTS2, COL14A1,PLOD2,COL6A3,PLG,MMP13,COL8A1,MMP9, TIMP1,COL5A2,COL17A1
**Syndecan 1 pathway (PID)**	2.191	<0.001	<0.001	COL1A1,COL12A1,COL3A1,COL1A2,COL5A1,COL6A1, COL6A2,CCL5,COL16A1,COL4A1,SDC1,COL14A1, COL6A3,COL8A1,MMP9,COL5A2,COL17A1
**AVB3 integrin pathway (PID)**	2.179	<0.001	<0.001	COL1A1,COL12A1,COL3A1,COL1A2,COL5A1,COL6A1, COL6A2,COL16A1,FN1,BCAR1,COL4A1,GPR124,SDC1, COL14A1,COL6A3,SRC,CSF1,COL8A1,COL5A2,COL17A1, VCL,RPS6KB1,COL2A1,EDIL3,RHOA
**Signaling by PDGF (Reactome)**	2.139	<0.001	<0.001	COL1A1,THBS2,COL3A1,PDGFRA,COL1A2,COL5A1, COL6A1,COL6A2,COL4A2,ADCY3,BCAR1,COL4A1, PDGFC,COL6A3,SRC,TRIB3,PLG,FOXO3,COL5A2,PLAT, STAT5A,ADCY6,GSK3A,THEM4,MAP2K1,COL2A1, PDGFRB,PDE1A,COL9A1,THBS1,RAPGEF1
**ECM receptor interaction (KEGG)**	2.114	<0.001	0.001	ITGA11,COL1A1,THBS2,COL3A1,SDC2,COL1A2,COL5A1, COL6A1,COL6A2,HSPG2,COL4A2,FN1,COL4A1,SDC1, COL6A3,TNC,ITGA9,LAMB2,LAMA4,COL5A2
**Integrin1 pathway (PID)**	2.096	<0.001	0.001	ITGA11,COL1A1,THBS2,COL3A1,FBN1,COL1A2,COL5A1, COL6A1,COL6A2,NID1,VCAM1,FN1,COL4A1,COL6A3, TNC,ITGA9,LAMB2,LAMA4,COL5A2
**Focal adhesion (KEGG)**	1.955	<0.001	0.017	ITGA11,COL1A1,THBS2,COL3A1,PDGFRA,COL1A2, COL5A1,COL6A1,COL6A2,CCND2,COL4A2,MYL9,FN1, BCAR1,MYLK,COL4A1,BRAF,PDGFC,COL6A3,TNC,SRC, EGFR,PAK3,PIK3R3,ITGA9,RAC3,LAMB2,LAMA4, COL5A2,FLT4,PARVG,VEGFC,VCL,MAPK8,DOCK1, VEGFB,FLNB,MAP2K1,SHC4,COMP,COL2A1,PDGFRB, VWF,RHOA,IBSP
**Integrin3 pathway (PID)**	1.933	<0.001	0.021	COL1A1,FBN1,COL1A2,CYR61,SPHK1,FN1,COL4A1, SDC1,TNC,LAMA4,PVR,EDIL3,PDGFRB,FGB,IBSP, THBS1,VEGFA
Smooth muscle contraction (Reactome)	1.915	<0.001	0.024	ACTG2,MYL9,CALD1,TPM2,MYLK,ACTA2,VCL
**Integrin cell surface interactions (Reactome)**	1.875	<0.001	0.041	ITGA11,COL1A1,FBN1,COL1A2,RAPGEF3,COL4A2, VCAM1,FN1,BCAR1,COL4A1,TNC,SRC,ITGAX,ITGA9, LAMB2,AMICA1,CDH1,COL2A1,VWF,FGB,IBSP, RAPGEF4
**NCAM1 interactions (Reactome)**	1.788	<0.001	0.112	COL1A1,COL3A1,COL1A2,COL5A1,COL6A1,COL6A2, COL4A2,COL4A1,COL6A3,NCAM1,COL5A2
Response to elevated platelet cytosolic Ca^{2+} (Reactome)	1.772	<0.001	0.126	KNG1,SERPING1,SPARC,TGFB3,FN1,PPBP,SERPINF2, TGFB2,PLG,TIMP1,VEGFC,VCL,VEGFB,VWF,FGB
**NCAM signaling for neurite out growth (Reactome)**	1.765	<0.001	0.125	COL1A1,COL3A1,COL1A2,COL5A1,COL6A1,COL6A2, COL4A2,COL4A1,COL6A3,SRC,NCAM1,COL5A2,GDNF, MAP2K1,COL2A1
Sphingolipid de novo biosynthesis (Reactome)	1.757	0.002	0.128	PPAP2A,SPHK1,SGMS2,PPAP2B,SPTLC2,COL4A3BP
Platelet activation signaling and aggregation (Reactome)	1.749	<0.001	0.131	KNG1,COL1A1,SERPING1,COL1A2,RAPGEF3,MGLL, SPARC,TGFB3,ADRA2A,FN1,BCAR1,PPBP,GNA15, SERPINF2,SRC,TGFB2,F2R,PLG,PIK3R3,LCK,TIMP1, VEGFC,VCL,VEGFB,GNAI1,DAGLA,GP5,VWF,FGB, RHOA,RAPGEF4,LAT,P2RY1,THBS1,DGKE,VEGFA, RASGRP1

Keratan sulfate biosynthesis (Reactome)	1.747	0.013	0.127	OGN,CHST1,ST3GAL2
Intrinsic pathway (Biocarta)	1.732	0.012	0.143	KNG1,SERPING1,COL4A2,COL4A1,F2R
A tetrasaccharide linker sequence is required for gag synthesis (Reactome)	1.717	0.003	0.160	DCN,SDC2,HSPG2,SDC1,GPC4,BCAN,BGN,B3GAT2, B4GALT7,GPC1
CXCR4 pathway (Biocarta)	1.708	0.007	0.165	CXCL12,BCAR1,MAP2K1,GNAI1

The pathways in bold involve ECM interactions; NES: Normalized enrichment score

Table S4. Upstream Regulators in Nur77-KO vs WT BMM

Upstream regulator	Molecule type	*Activation Z-Score	**p-Value of Overlap
KRAS	enzyme	-2.575	1.49E-06
DICER1	enzyme	-2.538	1.81E-02
NR4A3	ligand-dependent nuclear receptor	-2.224	4.47E-02
NR4A1	ligand-dependent nuclear receptor	-2.216	2.67E-03
CIITA	transcription regulator	-2.190	1.30E-03
POMC	peptide hormone	-2.051	8.92E-03
AHR	ligand-dependent nuclear receptor	-2.013	8.04E-05
IRF1	transcription regulator	-2.013	2.35E-01
PAX5	transcription regulator	-2.000	2.00E-02
MYOG	transcription regulator	2.000	2.97E-03
IL17RA	transmembrane receptor	2.000	2.00E-02
F2RL1	g-protein coupled receptor	2.000	6.23E-02
EGFR	kinase	2.097	1.19E-09
RAC1	enzyme	2.120	1.49E-06
CTNNB1	transcription regulator	2.140	4.31E-09
TCF3	transcription regulator	2.158	2.28E-03
F2R	g-protein coupled receptor	2.176	1.62E-04
TFEB	transcription regulator	2.200	6.75E-03
SMARCA2	transcription regulator	2.213	1.21E-03
F2	peptidase	2.219	6.34E-03
SEMA7A	transmembrane receptor	2.236	1.63E-03
VGLL3	transcription regulator	2.236	6.88E-05
TP53	transcription regulator	2.341	8.74E-07
ATF4	transcription regulator	2.369	6.75E-02
HIF1A	transcription regulator	2.407	1.55E-07
EGR1	transcription regulator	2.609	1.44E-07
SMARCA4	transcription regulator	2.615	2.87E-05
FOSL1	transcription regulator	2.745	4.81E-04
PRDM1	transcription regulator	2.758	2.62E-01
NR1I2	ligand-dependent nuclear receptor	3.123	2.73E-02
ERBB3	kinase	3.167	1.95E-07

*The bias-corrected z-score is used to infer the activation states of upstream regulators. It is calculated from the proportions of genes that are differentially regulated in an expected direction based on the known interactions between the regulator and the genes present in the Ingenuity database. Those genes with a z-score greater than 2 or less than -2 are considered to be either activated or inhibited respectively.

**The p-value of overlap is the calculated statistical significance of overlap between genes from the dataset and genes that are known to be regulated by the upstream regulator using Fisher's Exact Test.

Table S5. Top 25 up- and downregulated genes in LPS-stimulated Nur77-KO vs WT BMM

Gene Symbol	Log2 Fold Change (KO/WT)	p-value	Entrez Gene ID
Upregulated genes			
M34473	3.8122	0.00239	243469
Npy	3.7195	0.00972	109648
abParts	3.6913	0.00000	16019
abParts	3.3032	0.00401	16142
Slc11a2	2.5333	0.00000	18174
Eif2s3y	2.3580	0.05844	26908
Mxd1	1.9684	0.00163	17119
Ciita	1.9550	0.00353	12265
BC018473	1.9113	0.00175	193217
Gfpt1	1.7600	0.00008	14583
Serpinh1	1.7300	0.26895	12406
Kctd17	1.7261	0.00088	72844
R3hcc1	1.6868	0.00003	71843
Serping1	1.5740	0.15043	12258
Chi3l4	1.5691	0.00938	104183
Clic5	1.5597	0.00666	224796
Cxcl12	1.5385	0.13358	20315
Fnip1	1.5297	0.02440	216742
P4ha2	1.5282	0.09063	18452
Rragd	1.4409	0.00002	52187
abParts	1.4149	0.01144	16098
St6galnac2	1.4055	0.09316	20446
Actg2	1.3902	0.22570	11468
St5	1.3678	0.14188	76954
Nup210	1.3633	0.00088	54563
Downregulated genes			
Prpf39	-1.2123	0.01697	328110
Il16	-1.2125	0.02781	16170
Paqr9	-1.2398	0.01637	75552
Lrch4	-1.2447	0.02609	231798
Sla	-1.2568	0.05813	20491
Siglece	-1.2666	0.00123	83382
Shisa3	-1.2847	0.01924	330096
Rtn1	-1.2852	0.06569	104001
Ankrd37	-1.2955	0.02359	654824
Fmo3	-1.3048	0.00930	14262
Creb3l4	-1.3095	0.00275	78284
St7	-1.3286	0.04667	64213
Mdm1	-1.3384	0.00766	17245
Gm14378	-1.3524	0.01689	100044509
Gramd1a	-1.3585	0.00324	52857
Fos	-1.3700	0.00814	14281
Uchl1	-1.3708	0.04040	22223
Pou6f1	-1.4433	0.00068	19009
Bco2	-1.4619	0.00237	170752
Il6ra	-1.5065	0.00012	16194
1190003J15Rik	-1.5252	0.00421	76974
Jhdm1d	-1.5279	0.17590	338523
Gdf3	-1.5795	0.00002	14562
Anxa4	-1.6957	0.00003	11746
Il19	-1.8319	0.00134	329244

Table S6. Top 25 canonical pathways associated with differentially expressed genes in LPS-stimulated Nur77-KO vs WT BMM

IPA canonical pathway	p-value	Adjusted p-value	Ratio	z-score	Genes
Glucocorticoid Receptor Signaling	0.003	0.465	0.100	n.a	IFNG,CD3E,SGK1,IL10,HSPA1A/HSPA1B,PIK3R5,GTF2F1,CCL11,NFATC1,MED14,TGFBR2,FOS, TRAF2,POU2F1,POLR2A,NCOA2,DUSP1, SMARCA2,MAPK3,CREB1,HSP90AA1,NFATC2, PIK3R2,HLTF
Systemic Lupus Erythematosus Signaling	0.003	0.465	0.113	n.a	KNG1,CD3E,IL10,IL6R,PRPF39,PIK3R5FCGR2B, SART1,LSM2,NFATC1,CD28,FOS,LCK,MAPK3, CREM,NFATC2, PIK3R2
T Cell Receptor Signaling	0.005	0.465	0.128	n.a	FYN,FOS,CD28,LCK,CD3E,GRAP2,MAPK3, PIK3R5,NFATC2,PIK3R2,CD8BNFATC1
PKCθ Signaling in T Lymphocytes	0.006	0.465	0.120	-0.577	CD28,FOS,FYN,LCK,POU2F1,CD3E,HLA-DMA, GRAP2,MAPK3,PIK3R5,NFATC2,PIK3R2, NFATC1
CTLA4 Signaling in Cytotoxic T Lymphocytes	0.008	0.465	0.132	n.a	FYN,CD28,LCK,AP1G2,CD3E,GRAP2,CLTC, PIK3R5,PIK3R2,CD8B
Spermine and Spermidine Degradation I	0.009	0.465	0.667	n.a	SMOX,PAOX
Ascorbate Recycling (Cytosolic)	0.009	0.465	0.667	n.a	GSTO2,GLRX
L-serine Degradation	0.009	0.465	0.667	n.a	SRR,SDS
GDNF Family Ligand-Receptor Interactions	0.010	0.465	0.134	-1.667	FOS,DOK2,IRS1,MAPK3,CREB1,PIK3R5,IRS2, DOK3,PIK3R2
ILK Signaling	0.010	0.465	0.101	0.243	ITGB1,MYH10,FBLIM1,PIK3R5,VEGFB, CREB3L4,RICTOR,VEGFA,FOS,RHOT1,MAPK3, IRS1,CREB1,IRS2, ITGB4,PIK3R2,CTNNB1
Intrinsic Prothrombin Activation Pathway	0.014	0.557	0.185	n.a	KNG1,COL5A3,F9,KLK3,COL2A1
Role of BRCA1 in DNA Damage Response	0.020	0.682	0.127	1.000	IFNG,POU2F1,SMARCA2,RFC2,UIMC1RBL1, RFC5,HLTF
T Helper Cell Differentiation	0.020	0.682	0.127	n.a	TGFBR2,IFNG,CD28,IL10,IL12RB1, HLA-DMA, IL6R,BCL6
AMPK Signaling	0.022	0.685	0.102	-1.265	ADRA2B,TSC1,GNAS,PIK3R5,SMARCA2,IRS1, FASN,IRS2,PIK3R2, INSR,HLTF,ADRA1A,PPAT
Prostate Cancer Signaling	0.029	0.776	0.112	n.a	CCNE1,KLK3,MAPK3,CREB1,PIK3R5,HSP90AA1,CREB3L4,PIK3R2,CTNNB1
CD28 Signaling in T Helper Cells	0.032	0.776	0.102	-1.667	FYN,FOS,CD28,LCK,CD3E,GRAP2, HLA-DMA, PIK3R5,NFATC2,PIK3R2, NFATC1
Vitamin-C Transport	0.037	0.776	0.214	n.a	GSTO2,GLRX,TXNRD2
GDP-glucose Biosynthesis	0.038	0.776	0.333	n.a	HK1,PGM1
Role of Osteoblasts, Osteoclasts and Chondrocytes in Rheumatoid Arthritis	0.039	0.776	0.085	n.a	ITGB1,IFNG,IL10,PIK3R5,MMP13,WNT6, TNFRSF11A,IL7,CSF1R,XIAP,NFATC1,FOS, TRAF2,MAPK3,NFATC2,PIK3R2,CTNNB1, SMAD1
RANK Signaling in Osteoclasts	0.041	0.776	0.106	n.a	FOS,TRAF2,MAPK3,PIK3R5,NFATC2,PIK3R2, TNFRSF11A,XIAP,NFATC1
Prolactin Signaling	0.041	0.776	0.111	-0.378	FYN,FOS,IRS1,MAPK3,PIK3R5,NMI,PIK3R2,IRF1
iCOS-iCOSL Signaling in T Helper Cells	0.043	0.776	0.101	-1.414	GAB2,CD28,LCK,CD3E,GRAP2,HLA-DMA, PIK3R5,NFATC2,PIK3R2, NFATC1
Role of IL-17F in Allergic Inflammatory Airway Diseases	0.044	0.776	0.139	n.a	TRAF3IP2,MAPK3,CREB1,MMP13, CREB3L4
Regulation of IL-2 Expression in Activated and Anergic T Lymphocytes	0.050	0.776	0.107	n.a	TGFBR2,FYN,FOS,CD28,CD3E,MAPK3,NFATC2, NFATC1
Glucose and Glucose-1-phosphate Degradation	0.051	0.776	0.286	n.a	HK1,PGM1

Genes that are upregulated in Nur77-deficient BMMs are shown in red and green indicates that the expression of the gene is reduced upon Nur77 deficiency.

Z-score: a positive z-score indicates an overall increase in the activity of the pathway while a negative z-score predicts a decreased activity of that specific pathway when Nur77 is absent.

n.a., not applicable, meaning that it cannot be predicted based on the available literature whether the pathway is up- or downregulated.
