# Supplementary material for: Nur77-deficiency in bone marrow-derived macrophages modulates inflammatory responses, extracellular matrix homeostasis, phagocytosis and tolerance
Source: BMC Genomics. 2016 Mar 1;17:162. doi: 10.1186/s12864-016-2469-9 (PMC4774191; doi:10.1186/s12864-016-2469-9)
Supplement: Additional file 2: Figure S1. — Verification of the microarray results by qRT-PCR. mRNA expression of top differentially expressed genes, as determined by microarray analysis, cFos, S100A9, NPY and Serping1 in WT and Nur77-KO BMM was determined by qRT-PCR before (left panel) and after (right panel) treatment with 100 ng/ml LPS. Data are expressed as mean ± S.D. *p < 0.05, **p < 0.01, ***p < 0.001, ns = not significant. (PDF 311 kb) [file 12864_2016_2469_MOESM2_ESM.pdf]

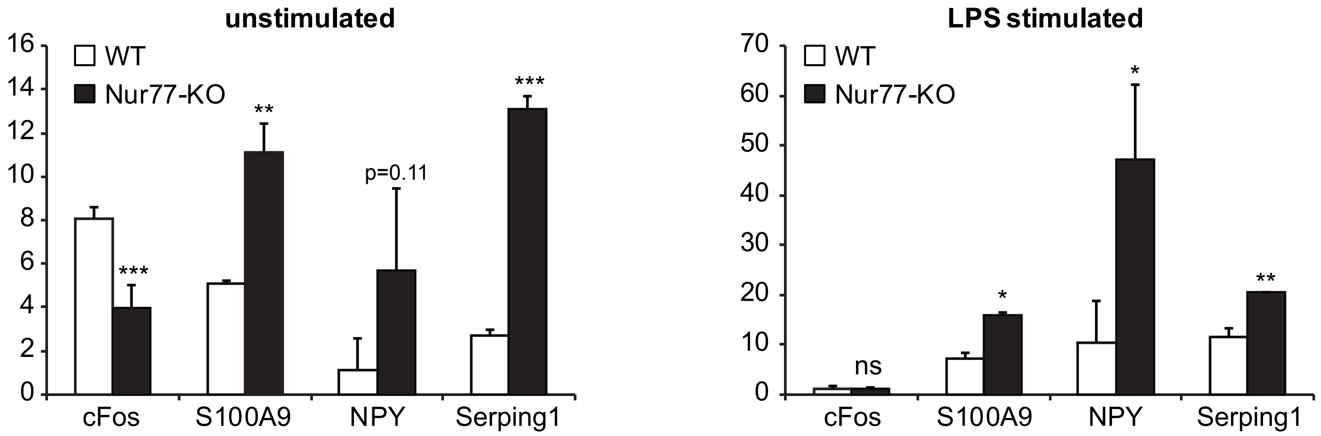

Figure S1. Verification of the microarray results by qRT-PCR.
mRNA expression of top differentially expressed genes, as determined by microarray analysis, cFos, S100A9, NPY and Serping1 in WT and Nur77-KO BMM was determined by qRT-PCR before (left panel) and after (right panel) treatment with 100 ng/ml LPS. Data are expressed as mean ± S.D. *p<0.05, **p< 0.01, ***p<0.001, ns=not significant.
